# *TERT-CLPTM1L* Polymorphism rs401681 Contributes to Cancers Risk: Evidence from a Meta-Analysis Based on 29 Publications

**DOI:** 10.1371/journal.pone.0050650

**Published:** 2012-11-30

**Authors:** Jieyun Yin, Yangkai Li, Ming Yin, Jingwen Sun, Li Liu, Qin Qin, Xiaorong Li, Lu Long, Shaofa Nie, Sheng Wei

**Affiliations:** 1 Department of Epidemiology and Biostatistics and MOE Key Lab of Environment and Health, School of Public Health, Tongji Medical College, Huazhong University of Science and Technology, Wuhan, China; 2 Department of Thoracic Surgery, Tongji Hospital, Tongji Medical College, Huazhong University of Science and Technology, Wuhan, China; 3 Department of Internal Medicine, Geisinger Medical Center, Danville, Pennsylvania, United States of America; Vanderbilt University, United States of America

## Abstract

**Background:**

Some common genetic variants of *TERT-CLPTM1L* gene, which encode key protein subunits of telomerase, have been suggested to play a crucial role in tumorigenesis. The *TERT-CLPTM1L* polymorphism rs401681 was of special interest for cancers risk but with inconclusive results.

**Methodology/Principal Findings:**

We performed a comprehensive meta-analysis of 29 publications with a total of 91263 cases and 735952 controls. We assessed the strength of the association between rs401681 and overall cancers risk and performed subgroup analyses by cancer type, ethnicity, source of control, sample size and expected power. Rs401681 C allele was found to be associated with marginally increased cancers risk, with per allele OR of 1.04 (95%CI = 1.00–1.08, *P*_heterogeneity_<0.001) and an expected power of 1.000. Following further stratified analyses, the increased cancers risk were discovered in subgroups of lung, bladder, prostate, basal cell carcinomas and Asians, while a declined risk of pancreatic cancer and melanoma were detected.

**Conclusions/Significance:**

These findings suggested that rs401681 C allele was a low-penetrance risk allele for the development of cancers of lung, bladder, prostate and basal cell carcinoma, but a potential protective allele for melanoma and pancreatic cancer.

## Introduction

Telomeres are repetitive (TTAGGG)n sequences into arrays of up to 25 kb and cap the end of linear chromosomes in human cells. They play a key role in counteracting the end-replication losses that occur as a consequence of semiconservative replication of linear DNA molecules [1], [2]. They also protect against coding sequence erosion and consequent DNA damage repair, which results in genome instability, chromosomal fusions, and rearrangements [3]. Telomerase and the control of telomere length are intimately linked to the process of tumourigenesis in humans. Telomerase have been showed to play a role in tumor progression and metasis by activation of the glycolytic pathway and suppression of cancer cell differentiation. Abnormal telomere length has been demonstrated in many cancers [4], [5].

5p15.33, which was commonly suggested to mediate the function of telomerase, contains two key genes: Telomerase reverse transcriptase (*TERT*) gene, and cleft lip and palate transmembrane 1 like gene (*CLPTM1L*; alias CRR9; MIM 612585). TERT is one of the main functional subunits of the telomerase enzyme and a key regulator of telomerase. Making use of the telomeric RNA subunit of telomerase as a template for the synthesis of single stranded DNA within the telomere, TERT thereby produce (TTAGGG)n tandem nucleotide repeats [6], [7]. TERT is reactivated in cancer cells. Mutations in the coding regions of *TERT* can affect telomerase activity and telomere length, and generate severe clinical phenotypes, including bone marrow failure syndromes and a substantive increase in cancer frequency [8]. Although the function of the *CLPTM1L* is largely unknown, studies have demonstrated that it may be involved in the apoptotic response to genotoxic stress induced by cisplatin, as it encodes a transcript whose over-expression has been shown to induce apoptosis in cisplatin-resistant-sensitive cells [9], [10], [11], [12]. Moreover, the *CLPTM1L* variants are hypothesized to enhance the metabolic activation of the reactive metabolites and/or formation and persistence of DNA adducts [13]. According to the latest dbSNP databse (http://www.ncbi.nlm.nih.gov/SNP), more than 3001 polymorphisms have been identified in the *TERT*-*CLPTM1L* locus. A series of cancer related single nucleotide polymorphisms (SNPs) have been reported in this region [14], [15], [16], [17]. A *TERT*-*CLPTM1L* SNP, rs401681 (C>T, located in the intron 13 of *CLPTM1L* and 27 kb from the *TERT* gene), is one of the most extensively studied SNPs. It has been reported to be associated with an increased risk of lung cancer through genome-wide association studies (GWAS) [18], [19], [20], [21], [22]. However, the reported genetic effects varied across the published studies. For example, an early study reported that the rs401681 T allele(the minor allele) was not associated with risk of lung cancer in 341 cases and 431 controls in Caucasians (*P*_trend_  = 0.259) [10], but another study with 2396 lung cancer cases and 3001 controls showed that the T allele was associated with a remarkably decreased risk of lung cancer in the same ethnicity (per allele OR  = 0.87; 95% CI  = 0.84–0.92) [23]. Additionally, a recent GWAS composed with 20726 cancer patients and 134650 controls suggested that the rs401681 C allele was associated with increased risk of lung, bladder, prostate and basal cell carcinomas [13]. More recently, the rs401681 C allele was inversely showed protective effect on melanoma risk in a study of 3843 cutaneous melanoma patients and 41963 controls [24], while another study did not find any significant association between rs401681 and risk of melanoma [25].

As above, the results remain controversial and ambiguous. Meanwhile, a single study might have been underpowered to detect the overall effects. A quantitative synthesis of the accumulated data from different studies is important to provide evidence on the association of rs401681 polymorphism with cancers risk. Thus, in this study, a comprehensive meta-analysis including the latest and relevant articles was conducted to explore whether rs401681 contribute to cancers risk.

## Materials and Methods

We conducted a systematic review and meta-analysis in accordance with the guidelines provided by the Human Genome Epidemiology Network [26].

### Search Strategy

A systematic literature searching was performed on PubMed, HuGE Navigator, Google Scholar and ISI Web of Science up to the end of July, 2012. The search strategy was based on combinations of “rs401681”, “*TERT*”, “telomerase reverse transcriptase,” “5p15.33,” “*CLPTM1L*,” or “*CLPTM1*-like”; “polymorphism,” “gene,” “variant”, “locus” or “SNP”; “association” or “risk”; “tumor”, “cancer”, “malignance”, “neoplasm” or “carcinoma”. In addition, references of the retrieved articles were scanned. Reviews, comments, and letters were also checked for additional studies.

### Inclusion and Exclusion Criteria

Articles which met the following criteria were included: (1) published in English; (2) the outcome was cancers; (3) tested for rs401681 polymorphism of *TERT-CLPTM1L* locus; (4) reported race and numbers of affected and unaffected subjects; (5) sufficient data for calculating an odds ratio (OR) with 95 percent confidence interval (95% CI) in additive model (two studies showed allelic ORs were also included because of large sample size and powerful influences [13], [24]).

Exclusion criteria were:(1) investigations in subjects with family cancer risks or cancer-prone disposition; (2) unpublished studies; (3) abstract, case report, comment, review and editorial; (4) Whenever reports pertained to overlapping patients, we retained only the largest study to avoid duplication of information.

### Data Extraction

The following information from each study was extracted by two investigators independently: (1) publication data, first author, year of publication; (2) cancer types; (3) ethnicity, source of control group and genotyping method; (4) minor allele frequency(MAF), genotype information (first priority) and/or additive OR and 95% CI, as additive model could recruit the largest number of subjects compared with any other genetic models in this meta-analysis; (5) for studies including subjects of different cancer types or ethnicities, data were extracted separately; (6) several included articles reported consortium or multistage results with multiple independent populations, if the summary OR did not show, these populations were listed as separated data sets. Discrepancies were resolved through discussion.

### Statistical Analysis

Deviation from the Hardy–Weinberg equilibrium (HWE) among controls subjects was tested by a χ^2^-test and a *P*<0.05 was considered as significant disequilibrium. The strength of the association between rs401681 polymorphism and overall cancers risk was measured by OR and corresponding 95%CI. Heterogeneity across studies was checked using the Cochran’s *Q*-test and considered significant at *P*<0.05 [27]. When homogeneity existed, the fixed model (Mantel-Haenszel method) was used to calculate the summary ORs and 95% CIs; otherwise, the random-effects model (the DerSimonian and Laird method) was utilized [27]. The quantity *I^2^* that presents the percentage of total variation across studies as a result of heterogeneity was also calculated [28].The potential source of heterogeneity among studies was explored by stratification and meta-regression analyses. Studies were categorized into different subgroups by type of cancer, ethnicity, source of control, sample size and expected power. If one cancer type contained less than three individual studies, it was combined into the “other cancers” group. When it comes to ethnicity, data sets were categorized as Caucasian, Asian and others. Meta-regression was performed to explore the source of heterogeneity among covariables,such as ethnicities, genotyping methods, source of controls, cancer types, sample size (<1000 and≥1000 subjects) and expected power (<0.5, 0.5 to 0.8 and>0.8) [29], [30]. Inverted funnel plots and the Egger’s test were used to examine publication bias [31]. Additionally, sensitivity analyses were performed by including and excluding studies not in HWE, and by removing sequential of individual studies. What’s more, we estimated the expected power of each individual study and subgroup analyses as determined by the probability of detecting a true association between rs401681 and cancers risk at the 0.05 level of significance, assuming OR of 1.2 (for a risk effect) or 0.83 (for a protective effect), with an alpha level equal to the observed *P* value [32]. All the *P* values were two sided, and all analyses were done in STATA statistical software (version10.0; Stata Corporation, college Station, Texas).

## Results

### Characteristics of All Included Studies

**Figure 1:**
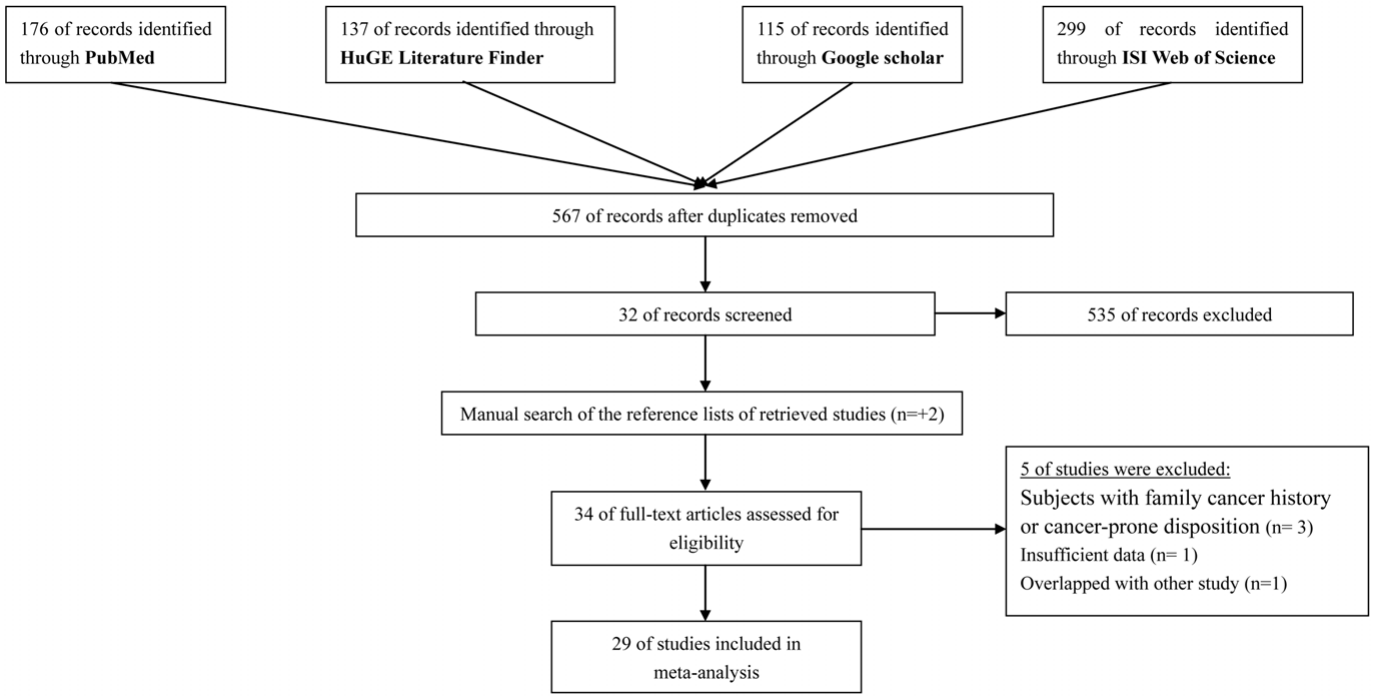
Literature search and study selection procedures for a meta-analysis of *TERT-CLTPM1L* polymorphism rs401681 and cancers risk.

As shown in **Figure 1**, 34 eligible original studies which through a comprehensive literature search up to the end of July, 2012, seemed to meet the inclusion criteria. However, after further examination, five studies were excluded because: three studies used subjects with family cancer history or cancer-prone disposition [33], [34], [35]; one study was overlapped with the study of Rafnar et al and had a smaller sample size [36], one study did not provide sufficient data [37]. Final data pool was consisted of 29 publications with 52 data sets. The research strategy was illustrated in **Figure 1** and **Table S1**.

As shown in **Table 1** and **Table S2**, 91263 cases and 735952 controls were relevant to the association between rs401681 and cancers risk [10], [13], [19], [22], [23], [24], [25], [26], [38], [39], [40], [41], [42], [43], [44], [45], [46], [47], [48], [49], [50], [51], [52], [53], [54], [55], [56], [57], [58]. SNPs in all studies were in agreement with HWE except one study [51]. Of these, there were 20 population-based or community-based data sets, 20 data sets with mixed controls, six hospital-based data sets, six data sets nested in cohort studies; one data set for cancers of cervical, glioblastoma multiforme, kidney, stomach, thyroid, lymphoma or multiple myeloma, two data sets of ovarian, colorectal, endometrial or testicular germ cell tumors; three data sets of breast, prostate or basal cell carcinomas; four data sets of pancreatic, squamous cell carcinomas or melanoma; five data sets of bladder cancer, eleven data sets of lung cancer. Four data sets were of small sample size (<1000 subjects) and 48 were of large sample size (≥1000 subjects). The number of data sets possessed low (<0.5), moderate (0.5 to 0.8) or high (>0.8) expected power were 9,12 and 31, respectively. Genotyping methods included Affymetrix, Illumina, MassARRAY, iPLEX and TaqMan and so on.

**Table 1:** Study Characteristics in an Analysis of the Association Between Rs401681 Polymorphism and Cancer Risk, 2008–2012.

Author, year of publication	Ethnicity	Genotypemethod	Sourceof control	Cancertype	No. ofCases/Controls	MAF	OR(95%CI	Expected Power
McKay:2008 [38]	Caucasian	TaqMan	Population	Lung Cancer	2971/3746	42.6	1.19(1.11–1.28)	0.589
Wang: 2008 [23]	Caucasian	Illumina & PCR-based KASPar	Mixed	Lung Cancer	7491/8721	40.7	1.15(1.09-1.19)	0.993
Rafnar:2009 [13]	Caucasian	Illumina	Mixed	Basal cell carcinoma	2565/29405	44.0	1.25(1.18–1.34)	0.125
	Caucasian	Illumina	Mixed	Lung cancer	4265/34666	44.0	1.15(1.10–1.22)	0.921
	Caucasian	Illumina	Mixed	Bladder cancer	4147/34988	46.5	1.12(1.06–1.18)	0.995
	Caucasian	Illumina	Mixed	Prostate cancer	9473/37901	44.7	1.07(1.03–1.11)	1.000
	Caucasian	Illumina	Population	Cervical cancer	369/28890	45.5	1.31(1.13–1.51)	0.113
	Caucasian	Illumina	Mixed	Breast cancer	3645/30030	44.2	0.98(0.94–1.02)	1.000
	Caucasian	Illumina	Mixed	Colorectal cancer	2495/29817	44.3	0.95(0.92–0.99)	1.000
	Caucasian	Illumina	Population	Melanoma	2443/30839	46.0	0.88(0.82–0.95)	0.933
	Caucasian	Illumina	Population	Endometrial cancer	470/28890	45.5	1.21(1.06–1.38)	0.451
	Caucasian	Illumina	Population	Kidney cancer	987/30722	44.2	1.08(0.97–1.19)	0.983
	Caucasian	Illumina	Population	Lymphoma	248/28890	45.5	0.87(0.72–1.05)	0.688
	Caucasian	Illumina	Population	Multiple myeloma	126/28890	45.5	1.21(0.93–1.58)	0.476
	Caucasian	Illumina	Population	Ovarian cancer	497/28890	45.5	0.98(0.84–1.14)	0.984
	Caucasian	Illumina	Population	Pancreatic cancer	301/28890	45.5	0.99(0.81–1.21)	0.957
	Caucasian	Illumina	Population	Squamous cell carcinoma	547/28890	45.5	1.14(1.00–1.30)	0.778
	Caucasian	Illumina	Population	Stomach cancer	762/28890	45.5	0.96(0.86–1.07)	0.996
	Caucasian	Illumina	Population	Thyroid cancer	528/28890	45.5	0.97(0.85–1.10)	0.992
Song:2009 [39]	Caucasian	Illumina	Population	Glioblastoma multiforme	149/149	40.9	0.90(0.61–1.34)	0.655
Stacey:2009 [24]	Caucasian	Illumina	Mixed	Basal cell carcinoma	3468/38107	45.0	1.20(1.13–1.27)	0.500
	Caucasian	Illumina	Population	Squamous cell carcinoma	1103/35824	45.0	1.04(0.94–1.16)	0.995
	Caucasian	Illumina	Mixed	Melanoma	3843/41963	45.0	0.86(0.81–0.91)	0.891
Zienolddiny:2009 [10]	Caucasian	TaqMan	Nested in cohort	Lung Cancer	341/431	46.9	1.20(0.98–1.47)	0.500
Gudmundsson:2010 [40]	Caucasian	Illumina	Mixed	Prostate cancer	1762/36375	42.2	1.07(0.86–1.33)	0.849
Kohno:2010 [41]	Asian	TaqMan	Mixed	Lung Cancer	2343/1173	33.4	1.14(1.01–1.25)	0.862
Liu: 2010 [42]	Caucasian	TaqMan	Hospital	SCCHN	1079/1115	45.2	1.05(0.93–1.18)	0.988
Miki:2010 [43]	Asian	Illumina	Population	Lung Cancer	1004/1900	31.9	1.17(1.04–1.31)	0.670
Petersen:2010 [22]	Mixed	Illumina	Mixed	Pancreatic Cancer	3532/3642	44.7	0.84(0.79–0.90)	0.633
Pooley:2010 [25]	Caucasian	TaqMan	Mixed	Breast Cancer	6800/6608	44.5	0.99(0.94–1.04)	1.000
	Caucasian	TaqMan	Mixed	Colorectal Cancer	2259/2181	45.0	0.98(0.90–1.06)	1.000
	Caucasian	TaqMan	Mixed	Melanoma	787/999	46.2	1.01(0.87–1.19)	0.980
Prescott:2010 [44]	Caucasian	TaqMan	Nested in cohort	Endometrial Cancer	674/1685	45.0	0.97(0.85–1.12)	0.981
Rothman:2010 [45]	Caucasian	Illumina	Mixed	Bladder Cancer	3526/5117	45.2	1.09(1.03,1.16)	0.999
Turnbull:2010 [46]	Caucasian	Illumina	Mixed	Testicular germ cell tumor	979/4947	44.4	0.79(0.71–0.87)	0.158
Yoon: 2010 [19]	Asian	Affymetrix &TaqMan & Invader assay	Population	Lung Cancer	1425/3011	32.1	1.26(1.15–1.40)	0.182
Beesley:2011 [47]	Caucasian	iPLEX	Mixed	Serous epithelial ovarian cancer	801/3247	43.0	0.90(0.80–1.01)	0.916
Gago-Dominguez:2011 [48]	Caucasian	TaqMan	Population	Bladder Cancer	472/554	44.2	1.18(0.98–1.41)	0.573
	Asian	TaqMan	Population	Bladder cancer	500/529	33.8	1.20(1.00–1.45)	0.500
Hu:2011 [49]	Asian	Affymetrix	Hospital	Lung cancer	2331/3077	32.3	1.10(1.01–1.19)	0.985
Kanetsky:2011 [26]	Caucasian	Affymetrix	Hospital	Testicular germ cell tumor	349/914	42.2	0.94(0.79–1.12)	0.918
Nan:2011 [50]	Caucasian	TaqMan	Nested in cohort	Melanoma	208/809	43.0	0.73(0.59–0.90)	0.115
	Caucasian	TaqMan	Nested in cohort	Squamous cell carcinoma	266/809	43.0	1.04(0.86–1.28)	0.912
	Caucasian	TaqMan	Nested in cohort	Basal cell carcinoma	283/809	43.0	1.12(0.92–1.35)	0.765
Pande:2011 [51]	Caucasian	Illumina	Hospital	Lung Cancer	1681/1235	45.3	1.28(1.15–1.43)	0.127
Rizzato:2011 [52]	Caucasian	PCR-based KASPar	Mixed	Pancreatic ductal adenocarcinoma	661/1267	42.5	0.85(0.74–0.97)	0.638
Bae:2012 [53]	Asian	PCR	Hospital	Lung cancer	1086/1079	31.3	1.07(0.94–1.22)	0.957
Chen:2012 [54]	Asian	TaqMan	Hospital	Lung cancer	195/228	27.9	0.98(0.73–1.32)	0.863
Klein,2012 [55]	Mixed	MassARRAY	Nested in cohort	Prostate cancer	943/2829	45.0	0.99(0.89–1.11)	0.999
Ma:2012 [56]	Asian	MassARRAY	Community	Bladder cancer	184/962	33.0	1.04(0.83–1.32)	0.880
Willis:2012 [57]	Caucasian	Illumina	Community	Pancreatic cancer	390/149	43.0	0.82(0.62–1.10)	0.468
Zheng:2012 [58]	African	Illumina	Mixed	Breast cancer	1509/1383	39.9	0.94(0.84,1.05)	0.986

Abbreviations: PCR, polymerase chain reaction; SCCHN,Squamous cell carcinoma of the head and neck.

Among 52 data sets included in this meta-analysis, 41 were conducted in Caucasians, eight in Asians, one was in Africans and two were of mixed ethnicities. The MAF of control subjects were 44.69% in Caucasians, 32.24% in Asians, 39.90% in Africans, respectively.

### Association between the rs401681 and Overall Cancers Risk

Rather than using ORs adjusted by covariables, our estimations were based on the raw data when possible. To assess the effect of adjustment, summary effect of ORs with and without adjustment were compared. Although there were subtle differences between these two sources of ORs in each study, the pooled ORs (95%CI) were nearly identical (**Figure S1**). These differences were of low impact to the synthesis, which were also suggested by other researchers [59], [60], [61], [62]. Meanwhile, we tried to explore the gap between allelic and additive ORs, and found them were amazing close to each other (**Figure S2**). As a result, two researches, which only reported allelic ORs for rs401681, were also included to final meta-analysis [13], [24].

**Figure 2:**
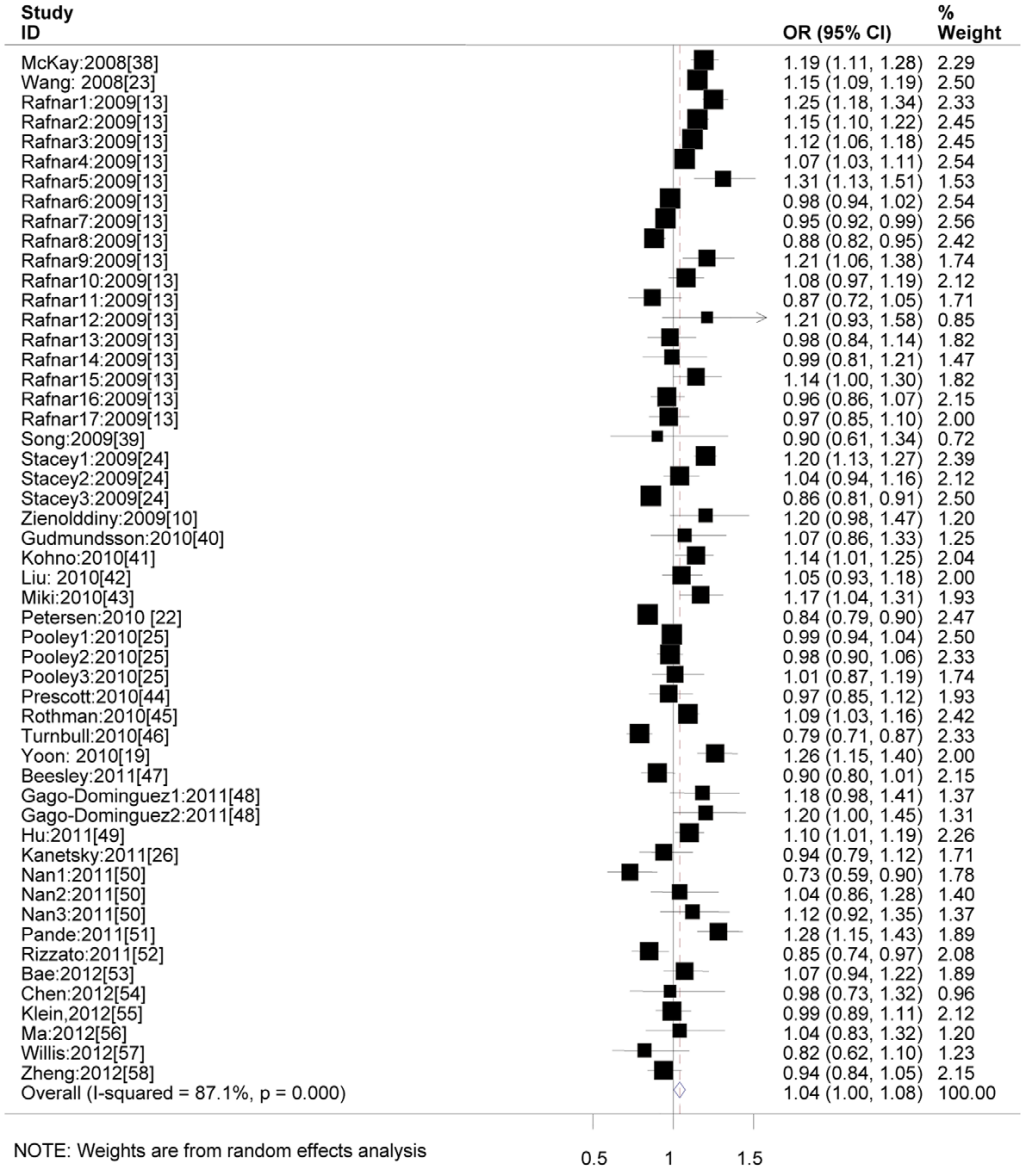
ORs of overall cancer risks associated with rs401681 under the additive model by random effects. For each data set, the OR and 95% CI was plotted with a box and a horizontal line. The symbol filled diamond indicates pooled OR and its 95% CI. Stacey1-3 represented studies for cancers of basal cell, squamous cell carcinomas and melanoma, respectively; Rafnar1-17 represented studies for basal cell, lung, bladder, prostate, cervical, breast, colorectal, melanoma, endometrial, kidney, lymphoma, multiple myeloma, ovarian, pancreatic, squamous cell, stomach and thyroid, respectively; Gago-Dominguez1-2 represented studies for bladder cancer in Caucasians and Asians, respectively; Pooley1-3 represented studies for breast, colorectal cancers and melanoma, respectively; Nan1-3 represented studies for melanoma, squamous cell and basal cell carcinomas, respectively.

As shown in **Figure 2**, the overall meta-analysis showed that rs401681 allele C marginally increased overall cancers risk in additive model (OR = 1.04; 95%CI: 1.00–1.08; *P_heterogeneity_* <0.001 and *I* ^2^ = 87.1%), with an expected power of 1.000.

### Stratified Analyses

In the stratified analyses by cancer types, as shown in **Table 2**, it was found that individuals with the C allele genotypes had higher risk of lung cancer (per allele OR = 1.16, 95%CI = 1.13–1.19; *P*_heterogeneity_ = 0.364 and *I* ^2^ = 8.40%), bladder cancer (per allele OR = 1.11, 95%CI = 1.07–1.15; *P*_heterogeneity_ = 0.774 and *I* ^2^ = 0), basal cell carcinoma (per allele OR = 1.22, 95%CI = 1.17–1.27; *P*_heterogeneity_ = 0.436 and *I* ^2^ = 0) and prostate cancer (per allele OR = 1.06, 95%CI = 1.02–1.10; *P*_heterogeneity_ = 0.406 and *I* ^2^ = 0), but lower risks of melanoma (per allele OR = 0.87, 95%CI = 0.83–0.91; *P*_heterogeneity_ = 0.098 and *I* ^2^ = 52.4%) and pancreatic cancer (per allele OR = 0.85, 95%CI = 0.80–0.90; *P*_heterogeneity_ = 0.558 and *I* ^2^ = 0). Additionally, C allele also contributed to a marginally higher susceptibly of squamous cell carcinoma (per allele OR = 1.06; 95%CI: 1.00–1.13; *P*_heterogeneity_ = 0.737 and *I* ^2^ = 0). However, no significant association was observed for other cancers.

**Table 2:** Results from Stratified Analysis of the Rs401681 and Cancer Risk, 2008–2011.

Category	No. of			Random effectmodel		Fixed effectmodel		*Q*	*P* a	*I^2^ (%)*	t^b^	*P* b	Expected power
	data sets	cases	controls	OR(95%CI)	*P*	OR(95%CI)	*P*						
**Overall**	52	91263	735952	1.04(1.00–1.08)	0.028			395.50	<0.001	87.1	−0.10	0.923	1.000
**Cancer type**													
Lung Cancer	11	25133	59267			1.16(1.13–1.19)	<0.001	10.92	0.364	8.4	0.02	0.986	1.000
Bladder Cancer	5	8829	42150			1.11(1.07–1.15)	<0.001	1.79	0.774	0	0.62	0.578	1.000
Melanoma	4	7281	74610			0.87(0.83–0.91)	<0.001	6.30	0.098	52.4	0.02	0.988	0.970
Squamous Cell Carcinoma	4	2995	66638			1.06(1.00–1.13)	0.050	1.27	0.737	0	0.15	0.892	1.000
Pancreatic Cancer	4	4884	33948			0.85(0.80–0.90)	<0.001	2.07	0.558	0	0.81	0.501	0.793
Basal cell carcinoma	3	6316	68321			1.22(1.17–1.27)	<0.001	1.66	0.436	0	−0.79	0.576	1.000
Breast Cancer	3	11954	38021			0.98(0.95–1.01)	0.189	0.71	0.701	0	−1.37	0.402	1.000
Prostate Cancer	3	12178	77105			1.06(1.02–1.10)	0.002	1.80	0.406	0	−0.71	0.605	1.000
Other cancers	15	11693	275892	0.98(0.93–1.04)	0.505			49.70	<0.001	71.8	0.89	0.388	1.000
**Ethnicity**													
Caucasian	41	76211	716139	1.03(0.99–1.07)	0.094			322.82	<0.001	87.6	−0.41	0.686	1.000
lung cancer	5	16749	48799			1.17(1.13–1.20)	<0.001	3.59	0.465	0			1.000
Melanoma	4	7281	74610			0.87(0.83–0.91)	<0.001	6.30	0.098	52.4			0.980
Squamous Cell Carcinoma	4	2995	66638			1.06(1.00–1.13)	0.074	1.27	0.737	0			1.000
Pancreatic cancer	3	1352	30306			0.88 (0.78–0.97)	0.010	1.65	0.438	0			0.880
Bladder cancer	3	8145	40659			1.11(1.07–1.15)	<0.001	0.88	0.645	0			1.000
Basal cell carcinoma	3	6316	68321			1.22(1.17–1.27)	<0.001	1.66	0.436	0			1.000
other cancers	19	33373	386806	0.99(0.95–1.03)	0.619			73.36	<0.001	75.5			1.000
Asian	8	9068	11959			1.14(1.09–1.19)	<0.001	7.47	0.382	6.3	−0.85	0.428	0.990
Others	3	5984	7854	0.91(0.82–1.01)	0.076			7.11	0.029	71.9			0.958
**Sources of control**													
Mixed	20	66351	352537	1.02(0.96–1.07)	0.417			274.27	<0.001	93.1	−0.72	0.482	1.000
Population- orCommunity- based	20	15476	368395	1.07(1.00–1.14)	0.030			81.62	<0.001	76.7	−0.12	0.906	1.000
Hospital-based	6	6721	7648	1.08(1.00–1.17)	0.043			11.3	0.046	55.8	−1.03	0.36	0.995
Nested in cohort	6	2715	7372	0.99(0.87–1.11)	0.863			15.00	0.010	66.7	0.07	0.95	0.999
**Sample size**													
Small	4	1075	957			0.99(0.85–1.12)	0.873	4.98	0.173	39.8	−1.77	0.219	0.997
Large	48	90188	734995	1.04(1.00–1.08)	0.042			390.27	<0.001	88.0	0.02	0.981	1.000
**Expected power**													
Low	9	8213	126226	1.10(0.92–1.27)	0.194			116.43	<0.001	93.1	−0.98	0.358	0.882
Moderate	12	14176	108914	1.07(0.96–1.19)	0.212			108.38	<0.001	89.9	−0.06	0.951	0.983
High	31	68874	500812	1.02(0.98–1.05)	0.181			163.05	<0.001	81.6	−0.54	0.596	1.000

a *P* values were calculated from Cochran’s Q test;

b *t* and *P* values were calculated by Egger’s test.

In terms of subgroup analyses by ethnicity, the associations were significant in Asian populations (per allele OR = 1.14; 95%CI: 1.09–1.19; *P*_heterogeneity_ = 0.382 and *I* ^2^ = 6.30%), while it was bordline significant in Caucasians (per allele OR = 1.03; 95%CI: 0.99–1.07) with high heterogeneity (*Q* = 322.82, *P*<0.001; *I* ^2^ = 87.6%). Stratified analysis by cancer types was performed in Caucasians, the results for lung, melanoma, squamous cell, pancreatic; bladder and basal cell carcinomas were the same in Caucasians as that of the overall population because these studies were mostly conducted in Caucasians (**Table 2**).

Further analyses also showed marginally significant results in hospital-based, population or community-based studies and studies of large sample size or high expected power (**Table 2**).

### Evaluation of Heterogeneity

The source of heterogeneity across studies was explored among covariables, such as ethnicities, genotyping methods,source of controls, cancer types, sample size and expected power. Interesting, cancer types were found to contribute to the heterogeneity across the studies in the overall (**Table S3**) and subgroups meta-analyses of Caucasians (data not shown).

### Sensitivity Analyses

A one-way sensitivity analysis was conducted to assess the influence of each individual study on the combined OR, with each particular data set dropped at a time. A random-effect model was employed when heterogeneity was indicated. Stability of odds ratio estimates was confirmed for association between rs401681 and cancers risk (**Figure S3**). Meanwhile, after the omission of the study departure from HWE, the results did not alter notably (data not shown).

### Publication Bias

**Figure 3:**
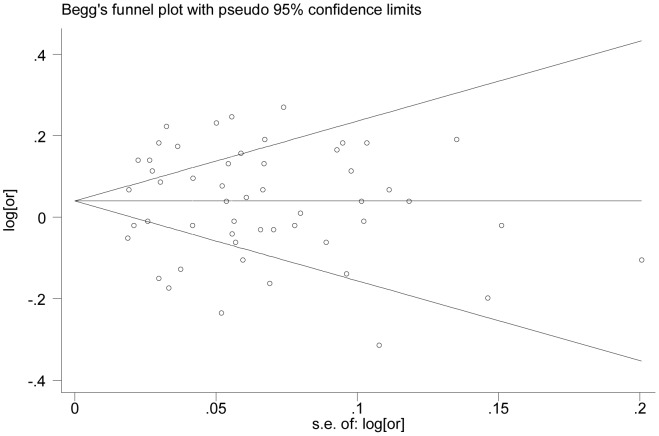
Funnel plot analysis to detect publication bias for the *TERT-CLMPT1L* polymorphism rs401681 in the involved 52 data sets.

Finally, funnel plots and the Egger’s test were used to assess publication bias. In the funnel plot analysis, the shape of the funnel plot seemed symmetrical (**Figure 3**). Furthermore, an Egger’s test did not detect any publication bias for rs401681 in the overall or subgroup analyses (**Table 2**). Therefore, there was no significant publication bias in the studies included in current analyses.

## Discussion

In the present meta-analysis, our results suggested that the carriers of rs401681 allele C had increased cancers risk, especially for lung, bladder, prostate and basal cell carcinomas, such effect was still found in subgroup of Asians; whereas decreased risk for melanoma and pancreatic cancer. Further exploration of the functional explanation of this locus is warranted to understand the mechanisms for these associations.

These findings have some degree of biological plausibility. The *TERT* gene is mapped to chromosome 5p15.33 and consists of 16 exons and 15 introns spanning 35 kb of chromosome 1. It encodes the catalytic subunit of telomerase, functions as telomere maintenance and may play a role in the determination of cancer risk [63]. TERT protein shows a high-level of expression in many tumors and it possibly contributes to unlimited cell division and carcinogenesis [64], [65]. The *CLPTM1L* gene has been documented to be upregulated in cisplatin-resistant cell lines and linked with cisplatin-induced apoptosis [9], and over-expression of *CLPTM1L* mRNA have been observed in many cancers [12], [13], [66]. Variants in this locus are hypothesized to mediate telomere length and be associated with multiple malignancies, including cancers of lung, prostate, urinary bladder, cervix and pancreas [13], [20], [21], [22], [67].

The heterogeneity among studies in this meta-analysis was dramatically reduced in stratified analyses by cancer types. It suggested a potential modified effect of *TERT-CLPTM1L* polymorphisms by tumor origins and the rational of stratified analyses. Therefore, we can infer that rs401681 had cancer-specific contributions and may play different roles in the etiology of different tumor sites [10], [19], [22], [23], [25], [26], [41], [42], [44], [48], [50], [68]. For example, strikingly increased risk was found in smoking related cancers, such as lung and bladder cancers. It may be explained that CLPTM1L protein may be involved in the apoptosis response of genotoxic stress [9], [10], [11], [12]. Further more, Nan and collaborators observed a suggestive positive relationship between rs401681 C allele and shorter relative telomere length [50]. Rafnar et al. suggested rs401681 C allele might be associated with an acceleration of the gradual shortening of telomeres with age [13]. Rs401681 was also demonstrated to be associated with risk of pancreatic cancer for chromosome ends lacking telomeric repeat sequences was observed in this cancer [69], [70]. Possible links between shorter telomeres and decreased risk of melanoma were reported [50], [67]. This might be because shorter telomere length conferring a shorter replicative lifespan in melanocyes, thus providing a more stringent barrier to unlimited cell division [67]. Declined melanoma risk might also due to the reduction of nevi size and count in individuals with shorter telomeres [67], [71]. Compared to the basal and squamous keratinocytes, melanocytes have a higher tendency to senescence in response to oncogenic stress, rather than undergoing cell apoptosis. In addition, shorter telomere length was reported to be associated with an increased risk of basal cell carcinoma [72]. This is probably suggested the different roles of replicative senescence in basal keratinocyets and melanocytes [72]. However, the exact biological function of rs401681 has not been clarified now. It may be in strong linkage disequilibrium (LD) with other potential functional or causal SNPs. For example,rs402710 located in the intron 15 of *CLPTM1L*,it is in strong LD with rs401681 (r^2^ = 0.70 in CEU, r^2^ = 0.89 in CHB and r^2^ = 0.759 in YRI), was predicted to have potential regulate function by SNPinfo and reported to be associated with higher levels of bulky aromatic and hydrophobic DNA adducts [10], [73]. Meanwhile, some SNPs in high LD with rs401681 are also predicted to have potential function by SNPinfo, including rs31490 (r^2^ = 0.910 in CEU, located in the transcription factor binding site of *CLPTM1L*) and rs414965 with high regulatory potential scores (located in the intron 11 of *CLPTM1L,* r^2^ = 0.759 in YRI) [73]. Further investigations were required to explore the role of rs401681 or SNPs in high LD with it in carcinogenesis, especially in various cancer types.

Furthermore, the different LD pattern of these potential functional SNPs with rs401681 in different ethnic populations may explain the different associations between rs401681 and cancer risk. Therefore, genetic backgrounds might explain, to some extent, the somewhat conflicting associations in different populations.

In terms of the control’s sources, the subtotal ORs and 95%CIs for rs401681 varied. The pooled ORs in population or community-based studies were 1.07(95%CI: 1.00–1.14) in additive model with an expected power of 1.000. Thus, these findings emphasized that the advantages of population based studies, including greater efficiency in sample recruitment, external validity than other study designs [74], [75].

There were still some limitations which need to be addressed. First, although no any publication bias was showed in the funnel plot and Egger’s tests, selection bias might still exist as non-English literatures were excluded. Second, the number of published studies was still insufficiently for the subgroup analyses of some particular cancer sites, such as endometrial, colorectal and testicular germ cell carcinomas. It might mask or exaggerate possible true associations. Third, due to insufficient genotype frequencies, we were unable to calculate the pooled ORs in other genetic models except additive model. Four, ORs with and without adjustment were pooled together, although there was no substantial changes between these two kinds of ORs in this synthesis, it might be a consideration source of heterogeneity. However, after restricting to crude estimations, only 24 data sets were available for synthesis. It might be non-representative and bias, which could lead to vastly different conclusions [76]. Additionally, matching was often assumed to account for confounders in case-control studies (the majority of studies were designed as case-control studies and had matched cases and controls in this meta-analysis). Thus, unadjusted findings represent adjustment that was accounted for by study design and not by the statistical methods [61]. However, we placed more emphasis on assessing biases across studies and tried to reduce potential sources of heterogeneity via stratification and sensitivity analyses. High expected powers of significant findings in this meta-analysis revealed great noteworthy and robustness. In view of this, we were confident that the findings in this meta-analysis were reliable and reasonable.

In conclusion, cumulated evidence suggested that rs401681 C allele was a tumor susceptibility allele in the development of lung, bladder, prostate and basal cell carcinomas, but a potential protective allele for pancreatic cancer and melanoma. These results suggested that the *TERT-CLTMP1L* polymorphism rs401681 may be potential biomarkers of cancer susceptibility. However, the effect on cancers risk may be modified by ethnicity, cancer type, source of controls and sample size. Future studies are required to validate the current findings.

## Supporting Information

Figure S1 **Additive ORs and corresponding 95%CI with and without adjustment were nearly identical for rs401681.** Gago-Dominguez1-2 represented studies for bladder cancer in Caucasians and Asians, respectively; Nan1-3 represented studies for melanoma, squamous cell and basal cell carcinomas, respectively.
(TIF)

Figure S2 **Additive ORs (95%CI) and corresponding allelic ORs (95%CI) of each data set for rs401681.** Gago-Dominguez1-2 represented studies for bladder cancer in Caucasians and Asians, respectively; Nan1-3 represented studies for melanoma, squamous cell and basal cell carcinomas, respectively.
(TIF)

Figure S3 **One-way sensitivity analyses**. The pooled odds ratios were calculated by omitting each data set at a time.
(TIF)

Table S1 **Search Strategies (Searched on 2012-07-20).**
(DOCX)

Table S2 **Genotype frequencies and Per Allele OR (95%CI) of Each Data Set enrolled.**
(DOCX)

Table S3 **Meta-regression to explore the source of heterogeneity in 52 included data sets.**
(DOCX)

## References

[1] Olovnikov AM (1971) [Principle of marginotomy in template synthesis of polynucleotides]. Dokl Akad Nauk SSSR 201: 1496–1499. 5158754

[2] Chudnovsky Y, Adams AE, Robbins PB, Lin Q, Khavari PA (2005) Use of human tissue to assess the oncogenic activity of melanoma-associated mutations. Nat Genet 37: 745–749. 15951821 10.1038/ng1586 PMC3063773

[3] Pooley KA, Sandhu MS, Tyrer J, Shah M, Driver KE, et al (2010) Telomere length in prospective and retrospective cancer case-control studies. Cancer Res 70: 3170–3176. 20395204 10.1158/0008-5472.CAN-09-4595 PMC2855947

[4] Greider CW, Blackburn EH (1985) Identification of a specific telomere terminal transferase activity in Tetrahymena extracts. Cell 43: 405–413. 3907856 10.1016/0092-8674(85)90170-9

[5] Bodnar AG, Ouellette M, Frolkis M, Holt SE, Chiu CP, et al (1998) Extension of life-span by introduction of telomerase into normal human cells. Science 279: 349–352. 9454332 10.1126/science.279.5349.349

[6] De Vivo I, Prescott J, Wong JY, Kraft P, Hankinson SE, et al (2009) A prospective study of relative telomere length and postmenopausal breast cancer risk. Cancer Epidemiol Biomarkers Prev 18: 1152–1156. 19293310 10.1158/1055-9965.EPI-08-0998 PMC2732000

[7] Zee RY, Castonguay AJ, Barton NS, Buring JE (2009) Mean telomere length and risk of incident colorectal carcinoma: a prospective, nested case-control approach. Cancer Epidemiol Biomarkers Prev 18: 2280–2282. 19661087 10.1158/1055-9965.EPI-09-0360 PMC2774215

[8] Baird DM (2010) Variation at the TERT locus and predisposition for cancer. Expert Rev Mol Med 12: e16. 20478107 10.1017/S146239941000147X

[9] Yamamoto K, Okamoto A, Isonishi S, Ochiai K, Ohtake Y (2001) A novel gene, CRR9, which was up-regulated in CDDP-resistant ovarian tumor cell line, was associated with apoptosis. Biochem Biophys Res Commun 280: 1148–1154. 11162647 10.1006/bbrc.2001.4250

[10] Zienolddiny S, Skaug V, Landvik NE, Ryberg D, Phillips DH, et al (2009) The TERT-CLPTM1L lung cancer susceptibility variant associates with higher DNA adduct formation in the lung. Carcinogenesis 30: 1368–1371. 19465454 10.1093/carcin/bgp131

[11] Wang S, Hu C, Zhu J (2007) Transcriptional silencing of a novel hTERT reporter locus during in vitro differentiation of mouse embryonic stem cells. Mol Biol Cell 18: 669–677. 17151355 10.1091/mbc.E06-09-0840 PMC1783791

[12] Asakura T, Imai A, Ohkubo-Uraoka N, Kuroda M, Iidaka Y, et al (2005) Relationship between expression of drug-resistance factors and drug sensitivity in normal human renal proximal tubular epithelial cells in comparison with renal cell carcinoma. Oncol Rep 14: 601–607. 16077962

[13] Rafnar T, Sulem P, Stacey SN, Geller F, Gudmundsson J, et al (2009) Sequence variants at the TERT-CLPTM1L locus associate with many cancer types. Nat Genet 41: 221–227. 19151717 10.1038/ng.296 PMC4525478

[14] Hung RJ, McKay JD, Gaborieau V, Boffetta P, Hashibe M, et al (2008) A susceptibility locus for lung cancer maps to nicotinic acetylcholine receptor subunit genes on 15q25. Nature 452: 633–637. 18385738 10.1038/nature06885

[15] Landi MT, Chatterjee N, Yu K, Goldin LR, Goldstein AM, et al (2009) A genome-wide association study of lung cancer identifies a region of chromosome 5p15 associated with risk for adenocarcinoma. Am J Hum Genet 85: 679–691. 19836008 10.1016/j.ajhg.2009.09.012 PMC2775843

[16] Hsu CP, Hsu NY, Lee LW, Ko JL (2006) Ets2 binding site single nucleotide polymorphism at the hTERT gene promoter–effect on telomerase expression and telomere length maintenance in non-small cell lung cancer. Eur J Cancer 42: 1466–1474. 16737810 10.1016/j.ejca.2006.02.014

[17] Carpentier C, Lejeune J, Gros F, Everhard S, Marie Y, et al (2007) Association of telomerase gene hTERT polymorphism and malignant gliomas. J Neurooncol 84: 249–253. 17410334 10.1007/s11060-007-9378-3

[18] Truong T, Hung RJ, Amos CI, Wu X, Bickeboller H, et al (2010) Replication of lung cancer susceptibility loci at chromosomes 15q25, 5p15, and 6p21: a pooled analysis from the International Lung Cancer Consortium. J Natl Cancer Inst 102: 959–971. 20548021 10.1093/jnci/djq178 PMC2897877

[19] Yoon KA, Park JH, Han J, Park S, Lee GK, et al (2010) A genome-wide association study reveals susceptibility variants for non-small cell lung cancer in the Korean population. Hum Mol Genet 19: 4948–4954. 20876614 10.1093/hmg/ddq421

[20] Landi MT, Chatterjee N, Yu K, Goldin LR, Goldstein AM, et al (2009) A genome-wide association study of lung cancer identifies a region of chromosome 5p15 associated with risk for adenocarcinoma. Am J Hum Genet 85: 679–691. 19836008 10.1016/j.ajhg.2009.09.012 PMC2775843

[21] Shete S, Hosking FJ, Robertson LB, Dobbins SE, Sanson M, et al (2009) Genome-wide association study identifies five susceptibility loci for glioma. Nat Genet 41: 899–904. 19578367 10.1038/ng.407 PMC4501476

[22] Petersen GM, Amundadottir L, Fuchs CS, Kraft P, Stolzenberg-Solomon RZ, et al (2010) A genome-wide association study identifies pancreatic cancer susceptibility loci on chromosomes 13q22.1, 1q32.1 and 5p15.33. Nat Genet 42: 224–228. 20101243 10.1038/ng.522 PMC2853179

[23] Wang Y, Broderick P, Webb E, Wu X, Vijayakrishnan J, et al (2008) Common 5p15.33 and 6p21.33 variants influence lung cancer risk. Nat Genet 40: 1407–1409. 18978787 10.1038/ng.273 PMC2695928

[24] Stacey SN, Sulem P, Masson G, Gudjonsson SA, Thorleifsson G, et al (2009) New common variants affecting susceptibility to basal cell carcinoma. Nat Genet 41: 909–914. 19578363 10.1038/ng.412 PMC2973331

[25] Pooley KA, Tyrer J, Shah M, Driver KE, Leyland J, et al (2010) No association between TERT-CLPTM1L single nucleotide polymorphism rs401681 and mean telomere length or cancer risk. Cancer Epidemiol Biomarkers Prev 19: 1862–1865. 20570912 10.1158/1055-9965.EPI-10-0281 PMC2901592

[26] Kanetsky PA, Mitra N, Vardhanabhuti S, Vaughn DJ, Li M, et al (2011) A second independent locus within DMRT1 is associated with testicular germ cell tumor susceptibility. Hum Mol Genet 20: 3109–3117. 21551455 10.1093/hmg/ddr207 PMC3131044

[27] DerSimonian R, Laird N (1986) Meta-analysis in clinical trials. Control Clin Trials 7: 177–188. 3802833 10.1016/0197-2456(86)90046-2

[28] Higgins JP, Thompson SG, Deeks JJ, Altman DG (2003) Measuring inconsistency in meta-analyses. BMJ 327: 557–560. 12958120 10.1136/bmj.327.7414.557 PMC192859

[29] Thompson SG, Sharp SJ (1999) Explaining heterogeneity in meta-analysis: a comparison of methods. Stat Med 18: 2693–2708. 10521860 10.1002/(sici)1097-0258(19991030)18:20<2693::aid-sim235>3.0.co;2-v

[30] Thompson SG, Smith TC, Sharp SJ (1997) Investigating underlying risk as a source of heterogeneity in meta-analysis. Stat Med 16: 2741–2758. 9421873 10.1002/(sici)1097-0258(19971215)16:23<2741::aid-sim703>3.0.co;2-0

[31] Egger M, Davey Smith G, Schneider M, Minder C (1997) Bias in meta-analysis detected by a simple, graphical test. BMJ 315: 629–634. 9310563 10.1136/bmj.315.7109.629 PMC2127453

[32] Wacholder S, Chanock S, Garcia-Closas M, El Ghormli L, Rothman N (2004) Assessing the probability that a positive report is false: an approach for molecular epidemiology studies. J Natl Cancer Inst 96: 434–442. 15026468 10.1093/jnci/djh075 PMC7713993

[33] Liu P, Vikis HG, Lu Y, Wang Y, Schwartz AG, et al (2010) Cumulative effect of multiple loci on genetic susceptibility to familial lung cancer. Cancer Epidemiol Biomarkers Prev 19: 517–524. 20142248 10.1158/1055-9965.EPI-09-0791 PMC2846747

[34] Barrett JH, Iles MM, Harland M, Taylor JC, Aitken JF, et al (2011) Genome-wide association study identifies three new melanoma susceptibility loci. Nat Genet 43: 1108–1113. 21983787 10.1038/ng.959 PMC3251256

[35] Shimizu M, Kiyotani K, Kunitoh H, Kamataki T, Yamazaki H (2011) Different effects of TERT, TP63, and CYP2A6 polymorphism on individual risk of tobacco-related lung cancer in male Japanese smokers. Journal of Cancer Therapy 2: 690–697.

[36] Kiltie AE (2010) Common predisposition alleles for moderately common cancers: bladder cancer. Curr Opin Genet Dev 20: 218–224. 20153630 10.1016/j.gde.2010.01.002

[37] Helfand BT, Kan D, Modi P, Catalona WJ (2011) Prostate cancer risk alleles significantly improve disease detection and are associated with aggressive features in patients with a “normal” prostate specific antigen and digital rectal examination. Prostate 71: 394–402. 20860009 10.1002/pros.21253 PMC3089434

[38] McKay JD, Hung RJ, Gaborieau V, Boffetta P, Chabrier A, et al (2008) Lung cancer susceptibility locus at 5p15.33. Nature Genetics 40: 1404–1406. 18978790 10.1038/ng.254 PMC2748187

[39] Song W, Ruder AM, Hu L, Li Y, Ni R, et al (2009) Genetic epidemiology of glioblastoma multiforme: confirmatory and new findings from analyses of human leukocyte antigen alleles and motifs. PLoS One 4: e7157. 19774073 10.1371/journal.pone.0007157 PMC2742900

[40] Gudmundsson J, Besenbacher S, Sulem P, Gudbjartsson DF, Olafsson I, et al.. (2010) Genetic Correction of PSA Values Using Sequence Variants Associated with PSA Levels. Science Translational Medicine 2. 10.1126/scitranslmed.3001513 PMC3564581 21160077

[41] Kohno T, Kunitoh H, Shimada Y, Shiraishi K, Ishii Y, et al (2010) Individuals susceptible to lung adenocarcinoma defined by combined HLA-DQA1 and TERT genotypes. Carcinogenesis 31: 834–841. 20061363 10.1093/carcin/bgq003

[42] Liu Z, Li G, Wei S, Niu J, Wang LE, et al (2010) Genetic variations in TERT-CLPTM1L genes and risk of squamous cell carcinoma of the head and neck. Carcinogenesis 31: 1977–1981. 20802237 10.1093/carcin/bgq179 PMC2966556

[43] Miki D, Kubo M, Takahashi A, Yoon KA, Kim J, et al (2010) Variation in TP63 is associated with lung adenocarcinoma susceptibility in Japanese and Korean populations. Nat Genet 42: 893–896. 20871597 10.1038/ng.667

[44] Prescott J, McGrath M, Lee IM, Buring JE, De Vivo I (2010) Telomere length and genetic analyses in population-based studies of endometrial cancer risk. Cancer 116: 4275–4282. 20549820 10.1002/cncr.25328 PMC2978514

[45] Rothman N, Garcia-Closas M, Chatterjee N, Malats N, Wu X, et al (2010) A multi-stage genome-wide association study of bladder cancer identifies multiple susceptibility loci. Nat Genet 42: 978–984. 20972438 10.1038/ng.687 PMC3049891

[46] Turnbull C, Rapley EA, Seal S, Pernet D, Renwick A, et al (2010) Variants near DMRT1, TERT and ATF7IP are associated with testicular germ cell cancer. Nat Genet 42: 604–607. 20543847 10.1038/ng.607 PMC3773909

[47] Beesley J, Pickett HA, Johnatty SE, Dunning AM, Chen X, et al (2011) Functional polymorphisms in the TERT promoter are associated with risk of serous epithelial ovarian and breast cancers. PLoS One 6: e24987. 21949822 10.1371/journal.pone.0024987 PMC3174246

[48] Gago-Dominguez M, Jiang X, Conti DV, Castelao JE, Stern MC, et al (2011) Genetic variations on chromosomes 5p15 and 15q25 and bladder cancer risk: findings from the Los Angeles-Shanghai bladder case-control study. Carcinogenesis 32: 197–202. 21081471 10.1093/carcin/bgq233 PMC3026841

[49] Hu Z, Wu C, Shi Y, Guo H, Zhao X, et al (2011) A genome-wide association study identifies two new lung cancer susceptibility loci at 13q12.12 and 22q12.2 in Han Chinese. Nat Genet 43: 792–796. 21725308 10.1038/ng.875

[50] Nan H, Qureshi AA, Prescott J, De Vivo I, Han J (2011) Genetic variants in telomere-maintaining genes and skin cancer risk. Hum Genet 129: 247–253. 21116649 10.1007/s00439-010-0921-5 PMC3443196

[51] Pande M, Spitz MR, Wu X, Gorlov IP, Chen WV, et al (2011) Novel genetic variants in the chromosome 5p15.33 region associate with lung cancer risk. Carcinogenesis 32: 1493–1499. 21771723 10.1093/carcin/bgr136 PMC3179422

[52] Rizzato C, Campa D, Giese N, Werner J, Rachakonda PS, et al (2011) Pancreatic cancer susceptibility Loci and their role in survival. PLoS One 6: e27921. 22125638 10.1371/journal.pone.0027921 PMC3220706

[53] Bae EY, Lee SY, Kang BK, Lee EJ, Choi YY, et al.. (2012) Replication of results of genome-wide association studies on lung cancer susceptibility loci in a Korean population. Respirology. 10.1111/j.1440-1843.2012.02165.x 22404340

[54] Chen XF, Cai S, Chen QG, Ni ZH, Tang JH, et al (2012) Multiple variants of TERT and CLPTM1L constitute risk factors for lung adenocarcinoma. Genet Mol Res 11: 370–378. 22370939 10.4238/2012.February.16.2

[55] Klein RJ, Hallden C, Gupta A, Savage CJ, Dahlin A, et al (2012) Evaluation of multiple risk-associated single nucleotide polymorphisms versus prostate-specific antigen at baseline to predict prostate cancer in unscreened men. Eur Urol 61: 471–477. 22101116 10.1016/j.eururo.2011.10.047 PMC3269546

[56] Ma Z, Hu Q, Chen Z, Tao S, Macnamara L, et al.. (2012) Systematic evaluation of bladder cancer risk-associated single-nucleotide polymorphisms in a Chinese population. Mol Carcinog. 10.1002/mc.21932 22711262

[57] Willis JA, Olson SH, Orlow I, Mukherjee S, McWilliams RR, et al (2012) A replication study and genome-wide scan of single-nucleotide polymorphisms associated with pancreatic cancer risk and overall survival. Clin Cancer Res 18: 3942–3951. 22665904 10.1158/1078-0432.CCR-11-2856 PMC3568955

[58] Zheng Y, Ogundiran TO, Adebamowo C, Nathanson KL, Domchek SM, et al (2012) Lack of association between common single nucleotide polymorphisms in the TERT-CLPTM1L locus and breast cancer in women of African ancestry. Breast Cancer Res Treat 132: 341–345. 22134622 10.1007/s10549-011-1890-7 PMC3670987

[59] Palomaki GE, Melillo S, Bradley LA (2010) Association between 9p21 genomic markers and heart disease: a meta-analysis. JAMA 303: 648–656. 20159873 10.1001/jama.2010.118

[60] Koushik A, Platt RW, Franco EL (2004) p53 codon 72 polymorphism and cervical neoplasia: a meta-analysis review. Cancer Epidemiol Biomarkers Prev 13: 11–22. 14744727 10.1158/1055-9965.epi-083-3

[61] Voils CI, Crandell JL, Chang Y, Leeman J, Sandelowski M (2011) Combining adjusted and unadjusted findings in mixed research synthesis. J Eval Clin Pract 17: 429–434. 21040243 10.1111/j.1365-2753.2010.01444.x PMC3063329

[62] Harder T, Bergmann R, Kallischnigg G, Plagemann A (2005) Duration of breastfeeding and risk of overweight: a meta-analysis. Am J Epidemiol 162: 397–403. 16076830 10.1093/aje/kwi222

[63] Wick M, Zubov D, Hagen G (1999) Genomic organization and promoter characterization of the gene encoding the human telomerase reverse transcriptase (hTERT). Gene 232: 97–106. 10333526 10.1016/s0378-1119(99)00108-0

[64] Bagheri S, Nosrati M, Li S, Fong S, Torabian S, et al (2006) Genes and pathways downstream of telomerase in melanoma metastasis. Proc Natl Acad Sci U S A 103: 11306–11311. 16847266 10.1073/pnas.0510085103 PMC1544082

[65] Nosrati M, Li S, Bagheri S, Ginzinger D, Blackburn EH, et al (2004) Antitumor activity of systemically delivered ribozymes targeting murine telomerase RNA. Clin Cancer Res 10: 4983–4990. 15297398 10.1158/1078-0432.CCR-04-0134

[66] Colombo J, Fachel AA, De Freitas Calmon M, Cury PM, Fukuyama EE, et al (2009) Gene expression profiling reveals molecular marker candidates of laryngeal squamous cell carcinoma. Oncol Rep 21: 649–663. 19212623

[67] Han J, Qureshi AA, Prescott J, Guo Q, Ye L, et al (2009) A prospective study of telomere length and the risk of skin cancer. J Invest Dermatol 129: 415–421. 18668136 10.1038/jid.2008.238 PMC2632304

[68] McKay JD, Hung RJ, Gaborieau V, Boffetta P, Chabrier A, et al (2008) Lung cancer susceptibility locus at 5p15.33. Nat Genet 40: 1404–1406. 18978790 10.1038/ng.254 PMC2748187

[69] Gisselsson D, Jonson T, Petersen A, Strombeck B, Dal Cin P, et al (2001) Telomere dysfunction triggers extensive DNA fragmentation and evolution of complex chromosome abnormalities in human malignant tumors. Proc Natl Acad Sci U S A 98: 12683–12688. 11675499 10.1073/pnas.211357798 PMC60114

[70] Wu X, Amos CI, Zhu Y, Zhao H, Grossman BH, et al (2003) Telomere dysfunction: a potential cancer predisposition factor. J Natl Cancer Inst 95: 1211–1218. 12928346 10.1093/jnci/djg011

[71] Bataille V, Kato BS, Falchi M, Gardner J, Kimura M, et al (2007) Nevus size and number are associated with telomere length and represent potential markers of a decreased senescence in vivo. Cancer Epidemiol Biomarkers Prev 16: 1499–1502. 17627017 10.1158/1055-9965.EPI-07-0152

[72] Mooi WJ, Peeper DS (2006) Oncogene-induced cell senescence–halting on the road to cancer. N Engl J Med 355: 1037–1046. 16957149 10.1056/NEJMra062285

[73] Xu Z, Taylor JA (2009) SNPinfo: integrating GWAS and candidate gene information into functional SNP selection for genetic association studies. Nucleic Acids Res 37: W600–605. 19417063 10.1093/nar/gkp290 PMC2703930

[74] Hancock DB, Scott WK (2007) Population-based case-control association studies. Curr Protoc Hum Genet Chapter 1: Unit 1 17. 10.1002/0471142905.hg0117s52 18428402

[75] Szklo M (1998) Population-based cohort studies. Epidemiol Rev 20: 81–90. 9762511 10.1093/oxfordjournals.epirev.a017974

[76] Colditz GA, Burdick E, Mosteller F (1995) Heterogeneity in meta-analysis of data from epidemiologic studies: a commentary. Am J Epidemiol 142: 371–382. 7625401 10.1093/oxfordjournals.aje.a117644

