# Supplementary figures and images for: TERT-CLPTM1L Polymorphism rs401681 Contributes to Cancers Risk: Evidence from a Meta-Analysis Based on 29 Publications
Source: PLoS One. 2012 Nov 30;7(11):e50650. doi: 10.1371/journal.pone.0050650 (PMC3511286; doi:10.1371/journal.pone.0050650)

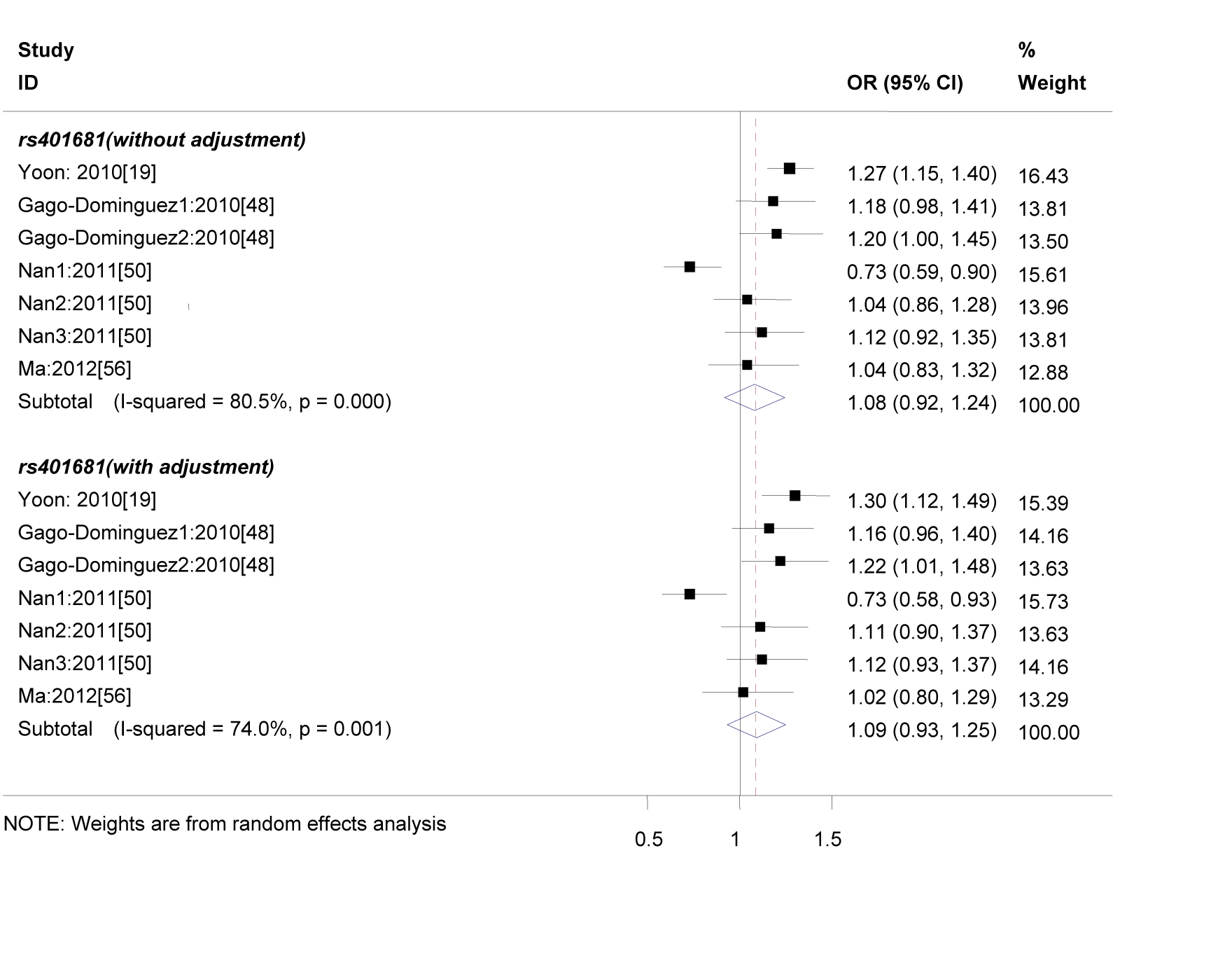

Supplement: Figure S1 — Additive ORs and corresponding 95%CI with and without adjustment were nearly identical for rs401681. Gago-Dominguez1-2 represented studies for bladder cancer in Caucasians and Asians, respectively; Nan1-3 represented studies for melanoma, squamous cell and basal cell carcinomas, respectively. (TIF) [file pone.0050650.s001.tif]

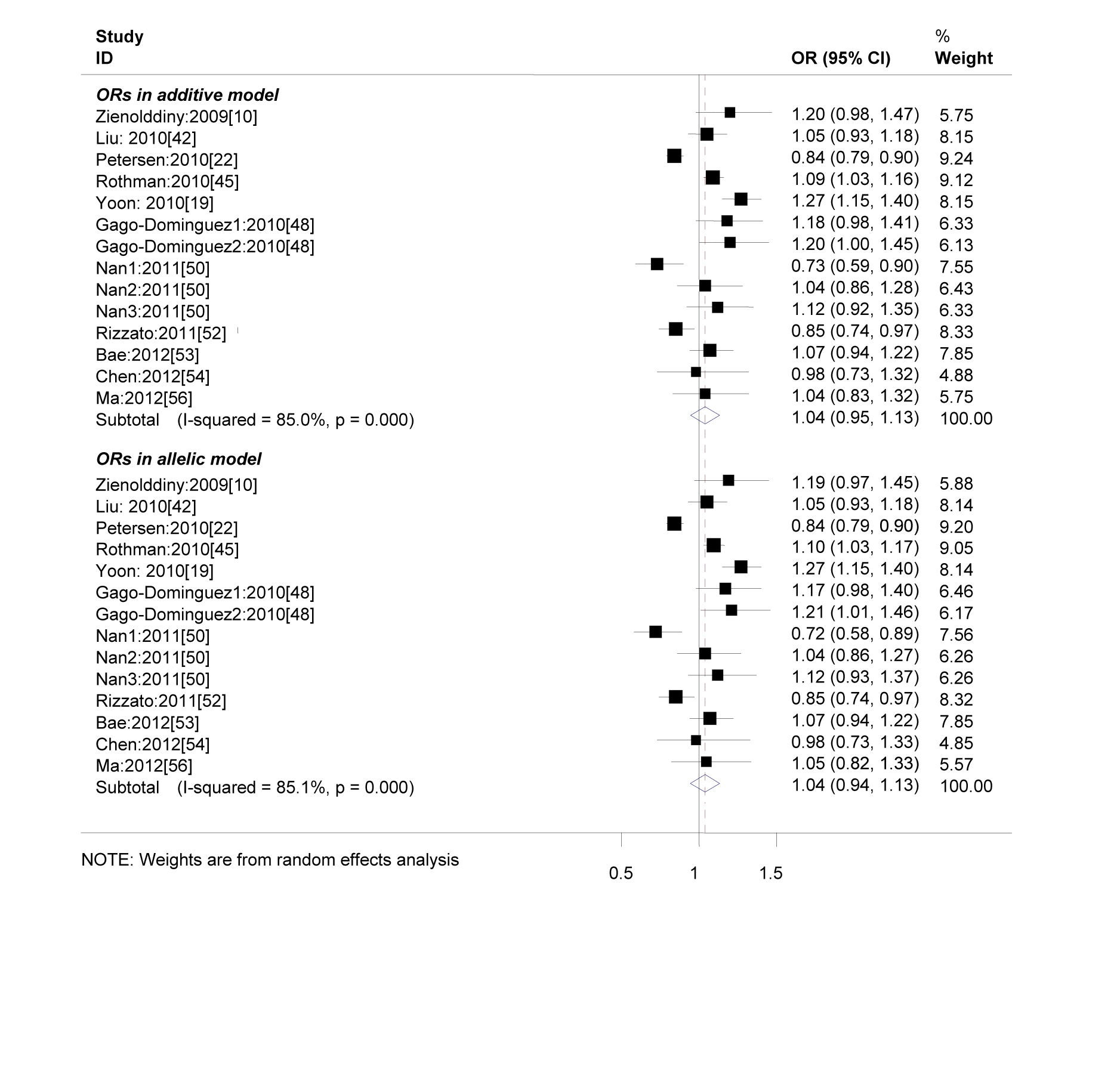

Supplement: Figure S2 — Additive ORs (95%CI) and corresponding allelic ORs (95%CI) of each data set for rs401681. Gago-Dominguez1-2 represented studies for bladder cancer in Caucasians and Asians, respectively; Nan1-3 represented studies for melanoma, squamous cell and basal cell carcinomas, respectively. (TIF) [file pone.0050650.s002.tif]

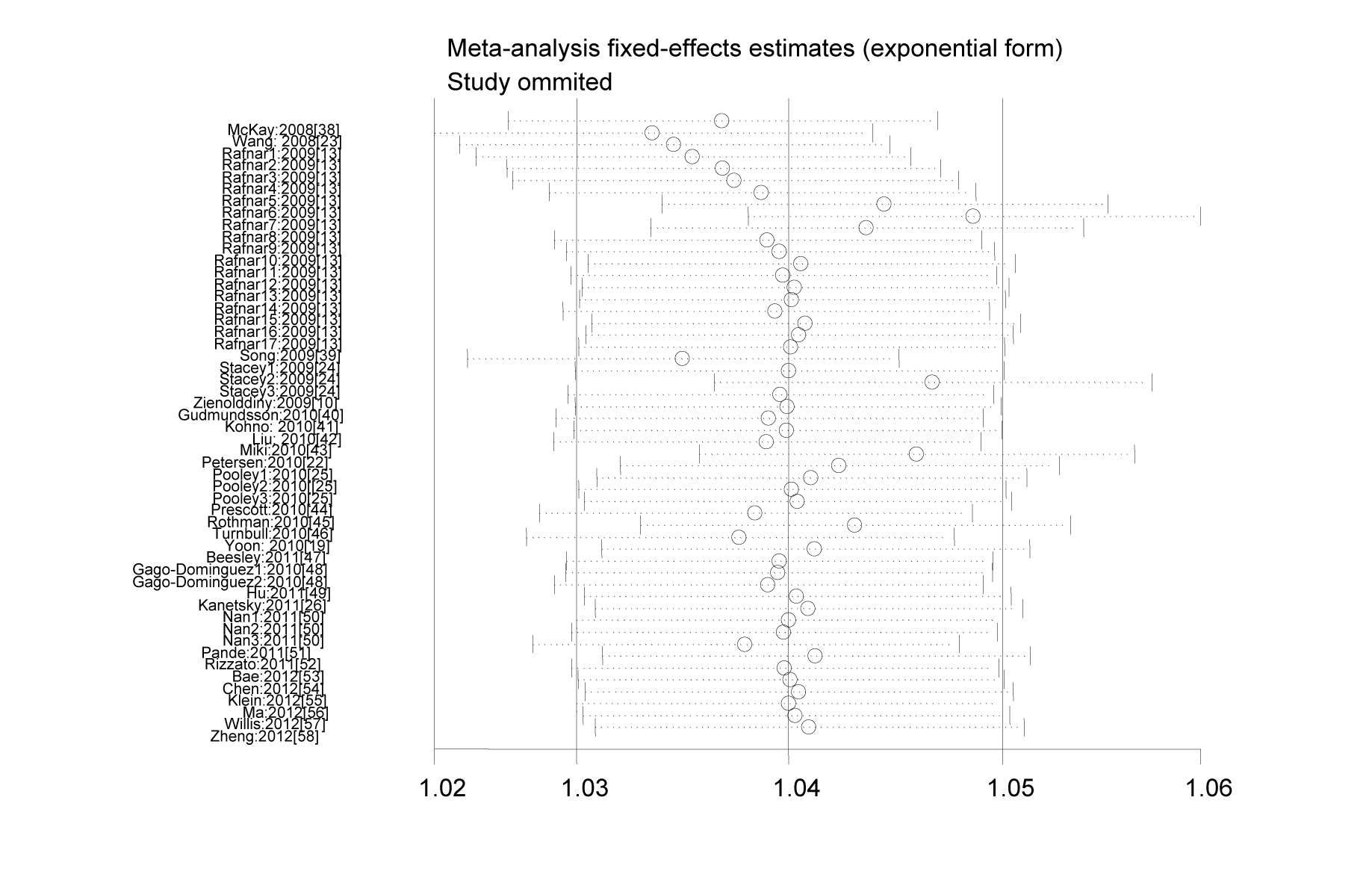

Supplement: Figure S3 — One-way sensitivity analyses. The pooled odds ratios were calculated by omitting each data set at a time. (TIF) [file pone.0050650.s003.tif]
