# Supplementary material for: TERT-CLPTM1L Polymorphism rs401681 Contributes to Cancers Risk: Evidence from a Meta-Analysis Based on 29 Publications
Source: PLoS One. 2012 Nov 30;7(11):e50650. doi: 10.1371/journal.pone.0050650 (PMC3511286; doi:10.1371/journal.pone.0050650)
Supplement: Table S1 — Search Strategies (Searched on 2012-07-20). (DOCX) [file pone.0050650.s004.docx]

| **Table S1.** Search Strategies (Searched on 2012-07-20) | | |  |
| --- | --- | --- | --- |
| **Database** |  | **Query** | **Items found** |
| **PubMed** |  | *(After duplicates removed, we got 176 of records in combination of #46 and #42)* |  |
|  | #46 | Pubmed (SNP Cited) for SNP (Select 401681) | 23 |
|  | #42 | (((#39) AND #38) AND #35) AND #34 Filters: Free full text available; Humans | 166 |
|  | #41 | (((#39) AND #38) AND #35) AND #34 Filters: Humans | 402 |
|  | #40 | (((#39) AND #38) AND #35) AND #34 | 461 |
|  | #39 | ((((tumor) OR cancer) OR malignance) OR neoplasm) OR carcinoma | 2950029 |
|  | #38 | (association) OR risk | 1852176 |
|  | #35 | ((((polymorphism) OR gene) OR variant) OR locus) OR SNP | 1829692 |
|  | #34 | (((((rs401681) OR TERT) OR telomerase reverse transcriptase) OR 5p15.33) OR CLPTM1L) OR CLPTM1-like | 31154 |
| **HuGE Navigator** |  |  |  |
|  |  | rs401681 OR TERT OR telomerase reverse transcriptase OR CLPTM1L OR CLPTM1-like OR 5P15.33 [TEXT+MESH] | 137 |
| **Google Scholar** |  |  |  |
|  |  | Rs401681, Language: English | 115 |
| **ISI Web of Science** |  |  |  |
|  | #5 | #4 AND #3 AND #2 AND #1  Document Types=( ARTICLE OR PROCEEDINGS PAPER ) AND Language=( ENGLISH )  Databases=SCI-EXPANDED, SSCI, A&HCI, CPCI-S, CPCI-SSH, CCR-EXPANDED, IC Time Span=All Years | 299 |
|  | #4 | Title=(polymorphism) OR Title=(gene) OR Title=(variant) OR Title=(locus) OR Title=(SNP)  Databases=SCI-EXPANDED, SSCI, A&HCI, CPCI-S, CPCI-SSH, CCR-EXPANDED, IC Time Span=All Years | 1651283 |
|  | #3 | Title=(association) OR Title=(risk)  Databases=SCI-EXPANDED, SSCI, A&HCI, CPCI-S, CPCI-SSH, CCR-EXPANDED, IC Time Span=All Years | 1676984 |
|  | #2 | Title=(tumor) OR Title=(cancer) OR Title=(malignance) OR Title=(neoplasm) OR Title=(carcinoma)  Databases=SCI-EXPANDED, SSCI, A&HCI, CPCI-S, CPCI-SSH, CCR-EXPANDED, IC Time Span=All Years | 1435116 |
|  | #1 | Title=(rs401681) OR Title=(TERT) OR Title=(telomerase reverse transcriptase) OR Title=(5p15.33) OR Title=(CLPTM1L) OR Title=(CLPTM1-like)  Databases=SCI-EXPANDED, SSCI, A&HCI, CPCI-S, CPCI-SSH, CCR-EXPANDED, IC Time Span=All Years | 44844 |
