# Supplementary material for: TERT-CLPTM1L Polymorphism rs401681 Contributes to Cancers Risk: Evidence from a Meta-Analysis Based on 29 Publications
Source: PLoS One. 2012 Nov 30;7(11):e50650. doi: 10.1371/journal.pone.0050650 (PMC3511286; doi:10.1371/journal.pone.0050650)
Supplement: Table S2 — Genotype frequencies and Per Allele OR (95%CI) of Each Data Set enrolled. (DOCX) [file pone.0050650.s005.docx]

| **Table S2.** Genotype frequencies and Per Allele OR (95%CI) of Each Data Set enrolled. | | | | | | | | | | | |
| --- | --- | --- | --- | --- | --- | --- | --- | --- | --- | --- | --- |
| **Author and Year**  **of publication** | **Genotype of**  **Cases** | | | **Genotype of**  **Controls** | | | **OR^a^** | **95%CI^a^** | **OR^bc^** | **95%CI^bc^** | **Note** |
|  | **CC** | **CT** | **TT** | **CC** | **CT** | **TT** |  |  |  |  |  |
| [McKay:2008](file:///C:\Users\yjy\AppData\Roaming\rs401681%201010.xlsx#RANGE!_ENREF_39)[[38](#_ENREF_38)] |  |  |  |  |  |  |  |  | 1.19 | 1.11-1.28 |  |
| Wang: 2008[[23](#_ENREF_23)] |  |  |  |  |  |  | 1.15 | 1.09-1.19 |  |  | GWAS discovery + replication phase |
| Rafnar^1^:2009[[13](#_ENREF_13)] |  |  |  |  |  |  |  |  | 1.25 | 1.18-1.34 |  |
| Rafnar^2^:2009[[13](#_ENREF_13)] |  |  |  |  |  |  |  |  | 1.15 | 1.10-1.22 |  |
| Rafnar^3^:2009[[13](#_ENREF_13)] |  |  |  |  |  |  |  |  | 1.12 | 1.06-1.18 |  |
| Rafnar^4^:2009[[13](#_ENREF_13)] |  |  |  |  |  |  |  |  | 1.07 | 1.03-1.11 |  |
| Rafnar^5^:2009[[13](#_ENREF_13)] |  |  |  |  |  |  |  |  | 1.31 | 1.13-1.51 |  |
| Rafnar^6^:2009[[13](#_ENREF_13)] |  |  |  |  |  |  |  |  | 0.98 | 0.94-1.02 |  |
| Rafnar^7^:2009[[13](#_ENREF_13)] |  |  |  |  |  |  |  |  | 0.95 | 0.92-0.99 |  |
| Rafnar^8^:2009[[13](#_ENREF_13)] |  |  |  |  |  |  |  |  | 0.88 | 0.82-0.95 |  |
| Rafnar^9^:2009[[13](#_ENREF_13)] |  |  |  |  |  |  |  |  | 1.21 | 1.06-1.38 |  |
| Rafnar^10^:2009[[13](#_ENREF_13)] |  |  |  |  |  |  |  |  | 1.08 | 0.97-1.19 |  |
| Rafnar^11^:2009[[13](#_ENREF_13)] |  |  |  |  |  |  |  |  | 0.87 | 0.72-1.05 |  |
| Rafnar^12^:2009[[13](#_ENREF_13)] |  |  |  |  |  |  |  |  | 1.21 | 0.93-1.58 |  |
| Rafnar^13^:2009[[13](#_ENREF_13)] |  |  |  |  |  |  |  |  | 0.98 | 0.84-1.14 |  |
| Rafnar^14^:2009[[13](#_ENREF_13)] |  |  |  |  |  |  |  |  | 0.99 | 0.81-1.21 |  |
| Rafnar^15^:2009[[13](#_ENREF_13)] |  |  |  |  |  |  |  |  | 1.14 | 1.00-1.30 |  |
| Rafnar^16^:2009[[13](#_ENREF_13)] |  |  |  |  |  |  |  |  | 0.96 | 0.86-1.07 |  |
| Rafnar^17^:2009[[13](#_ENREF_13)] |  |  |  |  |  |  |  |  | 0.97 | 0.85-1.10 |  |
| Song:2009[[39](#_ENREF_39)] |  |  |  |  |  |  | 0.90 | 0.61-1.34 |  |  |  |
| Stacey^1^:2009[[24](#_ENREF_24)] |  |  |  |  |  |  |  |  | 1.20 | 1.13-1.27 |  |
| Stacey^2^:2009[[24](#_ENREF_24)] |  |  |  |  |  |  |  |  | 1.04 | 0.94-1.16 |  |
| Stacey^3^:2009[[24](#_ENREF_24)] |  |  |  |  |  |  |  |  | 0.86 | 0.81-0.91 |  |
| [Zienolddiny:2009](file:///C:\Users\yjy\AppData\Roaming\rs401681%201010.xlsx#RANGE!_ENREF_10)[[10](#_ENREF_10)] | 107 | 177 | 57 | 117 | 224 | 90 | 1.20 | 0.98-1.47 |  |  |  |
| Gudmundsson:2010[[77](#_ENREF_77)] |  |  |  |  |  |  | 1.07 | 0.86-1.33 |  |  |  |
| [Kohno: 2010](file:///C:\Users\yjy\AppData\Roaming\rs401681%201010.xlsx#RANGE!_ENREF_46)[[41](#_ENREF_41)] |  |  |  |  |  |  |  |  | 1.14 | 1.01-1.25 |  |
| [Liu: 2010](file:///C:\Users\yjy\AppData\Roaming\rs401681%201010.xlsx#RANGE!_ENREF_59)[[42](#_ENREF_42)] | 357 | 495 | 227 | 333 | 557 | 225 | 1.05 | 0.93-1.18 |  |  |  |
| Miki:2010[[43](#_ENREF_43)] |  |  |  |  |  |  | 1.17 | 1.04-1.31 |  |  | Discovery phase |
| [Petersen:2010](file:///C:\Users\yjy\AppData\Roaming\rs401681%201010.xlsx#RANGE!_ENREF_58)[[22](#_ENREF_22)] |  |  |  |  |  |  |  |  | 0.84 | 0.79-0.90 |  |
| [Pooley^1^:2010](file:///C:\Users\yjy\AppData\Roaming\rs401681%201010.xlsx#RANGE!_ENREF_57)[[25](#_ENREF_25)] |  |  |  |  |  |  | 0.99 | 0.94-1.04 |  |  |  |
| [Pooley2:2010](file:///C:\Users\yjy\AppData\Roaming\rs401681%201010.xlsx#RANGE!_ENREF_57)[[25](#_ENREF_25)] |  |  |  |  |  |  | 0.98 | 0.90-1.06 |  |  |  |
| [Pooley3:2010](file:///C:\Users\yjy\AppData\Roaming\rs401681%201010.xlsx#RANGE!_ENREF_57)[[25](#_ENREF_25)] |  |  |  |  |  |  | 1.01 | 0.87-1.19 |  |  |  |
| [Prescott:2010](file:///C:\Users\yjy\AppData\Roaming\rs401681%201010.xlsx#RANGE!_ENREF_37)[[44](#_ENREF_44)] |  |  |  |  |  |  |  |  | 0.97 | 0.85-1.12 |  |
| Rothman:2010[[45](#_ENREF_45)] | 1126 | 1671 | 641 | 1521 | 2423 | 1042 | 1.09 | 1.03-1.16 |  |  | GWAS stage1 |
| Turnbull:2010[[46](#_ENREF_46)] |  |  |  |  |  |  | 0.79 | 0.71-0.87 |  |  | Discovery phase |
| [Yoon: 2010](file:///C:\Users\yjy\AppData\Roaming\rs401681%201010.xlsx#RANGE!_ENREF_52)[[19](#_ENREF_19)] | 751 | 574 | 100 | 1403 | 1283 | 325 | 1.27 | 1.15-1.40 | 1.30 | 1.12-1.49 |  |
| Beesley:2011[[47](#_ENREF_47)] |  |  |  |  |  |  |  |  | 0.90 | 0.80-1.01 |  |
| [Gago-Dominguez^1^:2010](file:///C:\Users\yjy\AppData\Roaming\rs401681%201010.xlsx#RANGE!_ENREF_53)[[48](#_ENREF_48)] | 164 | 235 | 73 | 170 | 278 | 106 | 1.18 | 0.98-1.41 | 1.16 | 0.96-1.4 |  |
| [Gago-Dominguez^2^:2010](file:///C:\Users\yjy\AppData\Roaming\rs401681%201010.xlsx#RANGE!_ENREF_53)[[48](#_ENREF_48)] | 248 | 207 | 45 | 237 | 226 | 66 | 1.20 | 1.00-1.45 | 1.22 | 1.01-1.48 |  |
| Hu:2011[[49](#_ENREF_49)] |  |  |  |  |  |  | 1.10 | 1.01-1.19 |  |  | Discovery phase |
| [Kanetsky:2011](file:///C:\Users\yjy\AppData\Roaming\rs401681%201010.xlsx#RANGE!_ENREF_20)[[26](#_ENREF_26)] |  |  |  |  |  |  |  |  | 0.94 | 0.79-1.12 | Discovery phase |
| [Nan1:2011](file:///C:\Users\yjy\AppData\Roaming\rs401681%201010.xlsx#RANGE!_ENREF_55)[[50](#_ENREF_50)] | 53 | 97 | 58 | 268 | 387 | 154 | 0.73 | 0.59-0.90 | 0.73 | 0.58-0.93 | Females only |
| [Nan2:2011](file:///C:\Users\yjy\AppData\Roaming\rs401681%201010.xlsx#RANGE!_ENREF_55)[[50](#_ENREF_50)] | 81 | 147 | 38 | 268 | 387 | 154 | 1.04 | 0.86-1.28 | 1.11 | 0.9-1.37 | Females only |
| [Nan3:2011](file:///C:\Users\yjy\AppData\Roaming\rs401681%201010.xlsx#RANGE!_ENREF_55)[[50](#_ENREF_50)] | 112 | 115 | 56 | 268 | 387 | 154 | 1.12 | 0.92-1.35 | 1.12 | 0.93-1.37 | Females only |
| Pande:2011[[51](#_ENREF_51)] |  |  |  |  |  |  |  |  | 1.28 | 1.15-1.43 |  |
| Rizzato:2011[[52](#_ENREF_52)] | 185 | 336 | 140 | 420 | 618 | 229 | 0.85 | 0.74-0.97 |  |  |  |
| Bae:2012[[53](#_ENREF_53)] | 545 | 434 | 107 | 499 | 484 | 96 | 1.07 | 0.94-1.22 |  |  |  |
| Chen:2012[[54](#_ENREF_54)] | 95 | 90 | 10 | 126 | 77 | 25 | 0.98 | 0.73-1.32 |  |  |  |
| Klein:2012[[55](#_ENREF_55)] |  |  |  |  |  |  | 0.99 | 0.89-1.11 |  |  |  |
| Ma:2012[[56](#_ENREF_56)] | 85 | 70 | 22 | 424 | 381 | 115 | 1.04 | 0.83-1.32 | 1.02 | 0.80-1.29 |  |
| Willis:2012[[57](#_ENREF_57)] |  |  |  |  |  |  | 0.82 | 0.62-1.10 |  |  |  |
| Zheng:2012[[58](#_ENREF_58)] |  |  |  |  |  |  |  |  | 0.94 | 0.84-1.05 |  |
| ^a^ Crude OR extracted from citation or OR calculated in Cochran-Armitage test as a dosage variant of 0,1,2 for increasing number of C allele;  ^b^ Adjusted OR and 95%CI in additive model that were extracted from citation;  ^c^ ORs derived from Simon N Stacey:2009[[24](#_ENREF_24)] and Rafnar:2009[[13](#_ENREF_13)] were calculated in allelic model;  Stacey1-3 represented studies for basal cell, squamous cell carcinomas and melanoma, respectively; Rafnar1-17 represented studies for basal cell, lung, bladder, prostate, cervical, breast, colorectal, melanoma, endometrial, kidney, lymphoma, multiple myeloma, ovarian, pancreatic, squamous cell, stomach, thyroid cancers, respectively; Gago-Dominguez1-2 represented studies for bladder cancer in Caucasians and Asians, respectively; Pooley1-3 represented studies for breast, colorectal cancers and melanoma, respectively; Nan1-3 represented studies for melanoma, squamous cell and basal cell carcinomas, respectively. | | | | | | | | | | | |
