# Supplementary material for: TERT-CLPTM1L Polymorphism rs401681 Contributes to Cancers Risk: Evidence from a Meta-Analysis Based on 29 Publications
Source: PLoS One. 2012 Nov 30;7(11):e50650. doi: 10.1371/journal.pone.0050650 (PMC3511286; doi:10.1371/journal.pone.0050650)
Supplement: Table S3 — Meta-regression to explore the source of heterogeneity in 52 included data sets. (DOCX) [file pone.0050650.s006.docx]

| **Table S3.** Meta-regression to explore the source of heterogeneity in 52 included data sets. | | | | | | |
| --- | --- | --- | --- | --- | --- | --- |
| **Covariant** | **Coef.** | **Std.Err.** | **t** | ***P*** | **95%Conf. Interval** | |
|  |  |  |  |  | **lower** | **upper** |
| Ethnicities | -0.0233973 | 0.0287187 | -0.81 | 0.420 | -0.0812397 | 0.0344452 |
| Genotyping methods | -0.007443 | 0.0122899 | -0.61 | 0.548 | -0.0321963 | 0.0173102 |
| Source of controls | 0.0123675 | 0.019794 | 0.62 | 0.535 | -0.0274997 | 0.0522346 |
| Cancer types | -0.0068273 | 0.0029901 | -2.28 | **0.027** | -0.0128497 | -0.000805 |
| Sample size | 0.1033678 | 0.0963706 | 1.07 | 0.289 | -0.0907325 | 0.2974681 |
| Expected Power | -0.0339457 | 0.0242602 | -1.40 | 0.169 | -0.0828082 | 0.0149169 |
| _cons | -0.0055998 | 0.2044469 | -0.03 | 0.978 | -0.417377 | 0.4061774 |
| Number of obs=52, tau2=0. 01183, I-squared_res=83.51%, Adj R-squared=11.63%, Model F(6,44)= 1.58, Prob > F=0.1749. | | | | | | |
